# Public preferences regarding data linkage for research: a discrete choice experiment comparing Scotland and Sweden

**DOI:** 10.1186/s12911-020-01139-5

**Published:** 2020-06-16

**Authors:** Mary P. Tully, Cecilia Bernsten, Mhairi Aitken, Caroline Vass

**Affiliations:** 1 Division of Pharmacy and Optometry, School of Health Sciences, Faculty of Biology, Medicine and Health, University of Manchester, Manchester Academic Health Science Centre, Manchester, UK; 2 Department of Public Health and Caring Sciences, Health and Medical Research, University of Uppsala, Uppsala, Sweden; 3 Newcastle University Business School, Newcastle University, Newcastle upon Tyne, UK; 4 Division of Population Health, Health Services Research & Primary Care, Faculty of Biology, Medicine and Health, University of Manchester, Manchester Academic Health Science Centre, Manchester, UK

**Keywords:** Preferences, discrete choice experiment, linked data

## Abstract

**Background:**

There are increasing examples of linking data on healthcare resource use and patient outcomes from different sectors of health and social care systems. Linked data are generally anonymised, meaning in most jurisdictions there are no legal restrictions to their use in research conducted by public or private organisations. Secondary use of anonymised linked data is contentious in some jurisdictions but other jurisdictions are known for their use of linked data. The publics’ perceptions of the acceptability of using linked data is likely to depend on a number of factors. This study aimed to quantify the preferences of the public to understand the factors that affected views about types of linked data and its use in two jurisdictions.

**Method:**

An online discrete choice experiment (DCE) previously conducted in Scotland was adapted and replicated in Sweden. The DCE was designed, comprising five attributes, to elicit the preferences from a representative sample of the public in both jurisdictions. The five attributes (number of levels) were: type of researcher using linked data (four); type of data being linked (four); purpose of the research (three); use of profit from using linked data (four); who oversees the research (four). Each DCE contained 6 choice-sets asking respondents to select their preferred option from two scenarios or state neither were acceptable. Background questions included socio-demographics. DCE data were analysed using conditional and heteroskedastic conditional logit models to create forecasts of acceptability.

**Results:**

The study sample comprised members of the public living in Scotland (*n* = 1004) and Sweden (*n* = 974). All five attributes were important in driving respondents’ choices. Swedish and Scottish preferences were mostly homogenous with the exception of ‘who oversees the research using linked data’, which had relatively less impact on the choices observed from Scotland. For a defined ‘typical’ linked data scenario, the probability (on average) of acceptance was 85.7% in Sweden and 82.4% in Scotland.

**Conclusion:**

This study suggests that the public living in Scotland and Sweden are open to using anonymised linked data in certain scenarios for research purposes but some caution is advisable if the anonymised linked data joins health to non-health data.

## Background

The process of producing linked datasets is defined as “the bringing together from two or more different sources, data that relate to the same individual, family, place or event” [1]. There are increasing examples of linking data on healthcare resource use and patient outcomes from different sectors of health and social care systems and potentially with data outside of health (linked data). For example, data from hospital admissions can be linked with medical records held by general practitioners (GPs) or more generally linking data from hospital medical records with national mortality data. Such linked data has been used to investigate the association between diabetes and cancer [2] or understand the hazards of discontinuing certain medications after an acute myocardial infarction [3]. It is also possible to link data from the health care sector with data from other sources, such as social care or education [4]. This broadens the types of research questions that can be addressed; for example, data from GP records linked with government data have been used to investigate the relationship between epilepsy diagnoses and social deprivation [5].

Research using linked data generally uses anonymised data, where data have been converted so that individuals can no longer be identified within the final dataset [6]. This process of producing anonymised linked data can occur before anonymization or by using a common identifier across the datasets comprising the linked data. In many jurisdictions, there are no legal restrictions to the use of such anonymised linked data, even in the absence of explicit patient consent. One example jurisdiction, Sweden, is famous for the extent of its use of national registries of anonymised linked data, and the universal unique personal identity number that makes data linkage comparatively straightforward [7, 8]. Despite the legality of using anonymised linked data, use of such data for purposes other than the original use for which the data were collected (‘secondary use’) has become contentious in some jurisdictions. Public objections have resulted in the failure of national data science initiatives in England [9] and Australia [10], for example.

There is also evidence of heterogeneity in views about the use of anonymised linked data within countries. Evidence suggests that some people are willing to give a general consent for using anonymised linked data but others are content not to be asked for consent provided that the data are used in studies that have been reviewed and approved by an ethics committee [11]. Published systematic reviews indicate the majority of the existing evidence on the preferences of the public about using anonymised linked data comes from the United Kingdom (UK) and particularly Scotland [12, 13]. Relatively little empirical research has been conducted to understand the preferences of the public from jurisdictions such as Sweden, which has widespread collection and use of anonymised linked data [7, 8]. Kodate conducted secondary analysis of media articles published in Swedish newspapers between 1995 and 2005 and identified that the media made frequent calls for improving the quality assurance systems underpinning the use and reporting of data from national registries [14].

Three published systematic reviews have summarised the literature that aims to understand the attitudes of members of the public to the use of linked data [12, 13, 15]. The individual studies identified in these systematic reviews were largely conducted in a single country, or a single region of a country, and there was a paucity of studies making a comparison between jurisdictions. There is some evidence that preferences may differ between jurisdictions. The European Commission’s Special Eurobarometer on Data Protection in 2011, reported that 66% of people living in Sweden were unconcerned about unnecessary disclosure of personal information compared with 19% of people living in the UK [16]. Furthermore, the majority (63%) of people living in Sweden were unconcerned about the secondary use of data compared with 20% of people living in the UK.

There are published examples of quantitative studies designed to collate views of the public about using anonymised linked data exist but these studies have traditionally used opinion-based survey or Likert-style agreement questions [13, 17–19], which are limited in their ability to identify the factors driving preferences. Questions which require rating or ranking may reveal individuals’ order of preference, but they are not able to quantify differences in magnitude of preference. For example, people may clearly prefer apples to oranges and on a traditional survey question may give apples all five marks and oranges only three marks, or rank apples before oranges in a list. From these rating, you can elicit the order of preference, but it is not always possible to understand how much better apples are than oranges, or how many oranges an individual would exchange for an apple. Similarly, it does not reveal when increases in apples are no longer satisfying to an individual; perhaps when the individual has 10 apples, they would prefer an orange.

Discrete choice experiments (DCEs) are a stated preference method which aim to elicit and quantify the preferences of a sample of the population for a specified service or product described by a set of characteristics (attributes and levels) [20]. A DCE takes account of the opportunity cost when making such choices as people have to make trade-offs between the attributes when they choose their preferred scenario from a set of hypothetical scenarios called ‘choice-sets’ [20]. In each choice-set, the respondent is presented with options described by the same attributes in varying amounts (levels) [21]. Under the theory of random utility maximisation (see Appendix), it is assumed that individuals choose the option which would provide them with the most value (‘utility’) and thus the choices made reveal both their preferences and the relative importance of each attribute when making their choice [22]. With the estimates of utility, is also possible to calculate the expected return and forecast from the collected data to estimate the probability of an individual choosing a particular scenario over another. DCEs are increasingly used to quantify people’s preferences for health goods, services and interventions, where normal markets rarely exist [23–26]. Although the number of studies in health economics is growing, applications started in the 1990s, making the approach relatively new compared to other question formats. Consensus-based guidelines on best research practice have been produced by the International Society for Pharmacoeconomics and Outcomes Research (ISPOR) for researchers seeking to use DCEs to quantify preferences in a healthcare context [27–29]. The aim of this study was to identify and compare the factors most influential in shaping public preferences, in two exemplar jurisdictions, for the type and use of linked data in a research context.

## Methods

This study used an online survey to field a DCE to compare the preferences of a sample of the public representing two jurisdictions (Scotland and Sweden). The Scottish DCE was conducted in 2016 [30]; this study replicated that DCE in Sweden, to enable comparisons between the two jurisdictions.

The DCE design and analysis are reported in line with published guidance [20, 29]. The online survey comprised four sections: an initial page of narrative introducing key concepts, and rationale for the sharing and use of anonymised linked data; questions about attitude to sharing and use of anonymised linked data; the choice-sets that formed the DCE; and socio-demographic questions.

### Conceptualising the choice question

**Fig. 1:**
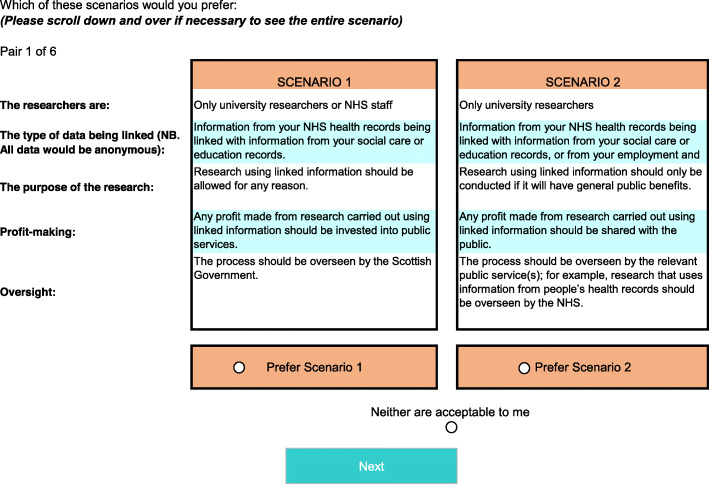
Example of scenario choice

The DCE used two ‘unlabelled’ alternatives to present scenarios describing the type and use of anonymised linked data. Respondents were asked to select which, if any, of the two alternatives was their preferred option. Respondents could also indicate if neither of the two alternatives was acceptable, allowing respondents the option to ‘opt-out’. Figure 1 shows an example choice-set.

### Attribute and level selection

Each alternative scenario was described with five attributes and plausible levels (see Table 1). For all but one attribute, there were four levels; the remaining attribute (research purpose) had three levels as there was no meaningful fourth level. Detail of the identification and generation of the five attributes and their levels has been published previously in relation to the original Scottish DCE [30]. Briefly, the attributes were chosen as the most important characteristics of sharing and using linked data of concern to the public, based on qualitative research [31] and a systematic review of the literature on public attitudes to linked data [12]. The levels were chosen to represent a range of actual or potential variations in these attributes and set to be within realistic and meaningful ranges to represent how linked data could be potentially shared and used. The wording of the attributes and levels was refined through iterations and engagement with members of an existing public involvement panel (the Farr Institute Scotland Public Panel).

**Table 1:** Attributes and Levels

Attribute	Levels (text variation for Sweden in brackets)
The researchers are:	Only university researchers.
	Only university researchers or NHS staff (researchers employed by a county council).
	Only university researchers, NHS staff or government researchers (researchers employed by a county council or researchers employed by one of the authorities).
	University researchers, NHS staff, government researchers (researchers employed by a county council or researchers employed by one of the authorities) and commercial researchers such as market research organisations or pharmaceutical companies.^a^
The type of data being linked:	Information from your GP (primary care) records being linked with information from your other NHS (county council) health records e.g. hospital records.
	Information from your NHS (county council) health records being linked with information from your social care or education records.
	Information from your NHS (county council) health records being linked with information from your social care or education records, or from your employment and benefits (national health insurance) records.
	Information from your NHS (county council) health records being linked with information from your social care, education, employment, and benefits (national health insurance) records, as well as information collected about you in the private sector e.g. through online shopping accounts. ^a^
The purpose of the research:	Research using linked information should only be conducted if it will have direct benefits for the people whose information is being used.
	Research using linked information should only be conducted if it will have general public benefits.
	Research using linked information should be allowed for any reason.^a^
Profit-Making:	Nobody should be allowed to profit from research carried out using linked information.
	Any profit made from research carried out using linked information should be shared with the public.
	Any profit made from research carried out using linked information should be invested into public services.
	Any profit made from research carried out using linked information should be kept by those carrying out the research. ^a^
Oversight:	The process should be overseen by the Scottish (Swedish) Government.
	The process should be overseen by a non-governmental independent body (an independent body that is not part of the Swedish Government).
	The process should be overseen by the relevant public service(s); for example, research that uses information from people’s health records should be overseen by the NHS (county council).
	The process should be overseen by the organisations undertaking the research. ^a^

^a^ base level in the analysis

### Experimental design

There were 768 (4^4^ × 3^1^) unique profiles possible from the chosen attributes and levels, which could create 294,528 different combinations for the choice-tasks. To reduce this unmanageable number of potential alternatives, a main effects design was generated using Sawtooth Software [32] with each respondent allocated to one of 40 blocks each containing six choice-sets. The paired alternatives selected by the software were reviewed to remove irrational or implausible choice sets. Pilot testing in Scotland revealed that using 12 choice-sets resulted in respondent fatigue, hence each respondent was presented with six choice-sets in a random order in the main survey [30].

### Survey design and piloting

The DCE was embedded as part of an online survey, as described earlier. The Swedish DCE used the same design as that used previously in Scotland, with appropriate changes for the different organisational health care systems. Forward and backward translation was conducted by an independent organisation and validated by bilingual members of the research team. Each survey was tested, using qualitative piloting in interviews in each country, with a convenience sample of 20 people of a variety of ages and gender. The aims of the qualitative pilot were to ensure respondents understood the instructions and the language used, and to test how they interacted with the survey and how long they took to complete it. Minor changes were made to the ordering and wording of some questions in Scotland for improved clarity [30] and these were carried forward to the Swedish survey where no additional changes were needed.

### Study population and sampling frame

The relevant study population for this study were adult (18 years and over) members of the public from two selected example jurisdictions (Scotland and Sweden). Scotland was chosen as an exemplar because National Health Service (NHS) Scotland is a publicly funded health care system that has the capacity to share and use anonymised linked data. Sweden was chosen as a comparator because the use of linked data is relatively more common, with large national registries integrating health and other social data used to answer a range of research questions. The two jurisdictions have comparable universal healthcare coverage by either national (the NHS in Scotland) or local (county councils in Sweden) providers, respectively.

For a DCE, the required sample size depends on the number of choice-sets, the number of alternatives in a choice set, and the number of levels attached to an attribute [33]. Given these characteristics, and the objectives to explore preference heterogeneity and compare the responses between Scotland and Sweden, a sample of 1000 respondents from each country was deemed more than sufficient for this study. In this DCE, the power calculation for sample size suggested by Orme would indicate a minimum sample size of 167 [33]. This power calculation, however, does not make allowances for investigations into preference heterogeneity nor the difference in preferences between Sweden and Scotland. A published review of sample sizes in DCEs found that, out of 505 healthcare DCE studies, only six had sample sizes of over 1000 [34].

The DCE was sent to a sample of adult members of the public in the two countries (Scotland and Sweden). The sample was identified using an international market research company, Ipsos [35] (called Ipsos Mori in the UK), who provide members of online panels [36]. Participants were members of the Ipsos international panel (called i-Say), who had volunteered to take part in regular market research surveys. Panellists received regular invitations from Ipsos to participate in surveys and were free to decide whether to complete any individual survey. Panellists were selected at random, and invited to take part via an email, with quotas set on key demographic variables, namely, age, gender, and working status, with the aim of achieving a sample of 1000 people in each country who were representative of the population for these criteria. The Scottish survey was conducted in August 2016 and the Swedish survey in June 2017; both were live for 14 days, until the quotas were filled.

Screening questions were used at the start of the survey, based on the attributes and levels. For example, respondents were asked which of the levels in the attribute “the purpose of research” was closest to their view, along with the option to select that “data linkage should not be permitted under any circumstances”. Respondents selecting the latter were routed-out and did not complete the DCE [30]. It was hypothesised that these respondents would always select the opt-out option. Removing them from the sample ensured that DCE respondents did not fundamentally object to data linkage and thus allowed an investigation of the nuanced public preferences for conducting research with linked data.

### Analysis

Choice data from the DCE were analysed using discrete choice models. All attributes were categorical and were dummy coded relative to a base level (Table 1) that was deemed to be the ‘worst’. The primary analysis estimated the preferences from each sample of respondents from the two countries separately using a conditional logit model. To further compare data between Scotland and Sweden, a pooled conditional logit model was estimated with interaction terms between dummy variables that identified the respondent’s nationality (1 = Scottish) and each attribute level. To account for differences in scale, a pooled heteroskedastic conditional logit model with these same interactions was also estimated [37, 38]. The scale parameter was allowed to vary by the respondent’s nationality. In order to identify the scale term, preferences over one attribute must be restricted to be equal across countries. This attribute (purpose of the research) was selected based on statistically insignificant interaction terms in the pooled conditional logit model. All analyses were completed using Stata 13 [39].

The probability of an individual finding a specific scenario acceptable was calculated by estimating the expected observable utility of an alternative and comparing it with expected utility of another. A ‘typical’ linked data scenario was defined as university researchers or health service staff using linked health records for general public benefit, the profit is invested in public services and the process is overseen by the relevant public services. Two scenarios were then specified as: best-case (the most risk averse scenario, where only university researchers use linked data from health records for the benefit of people whose data are being used, there is no profit made and the process is overseen by a non-governmental body) and worst-case (where university researchers, health service staff, government and commercial researchers use health data linked to social care, education, employment and private sector data for research with any purpose, where the profit is kept by those carrying out these research who also oversee the process). Investigations into preference heterogeneity were conducted using a split sample analysis and comparing the probabilities of scenarios being acceptable.

## Results

A total of 1978 respondents completed the survey and were included in the analysis (Table 2). An additional 974 respondents (461 in Scotland and 513 in Sweden) started the survey but were routed-out at the initial questions (presented in the order shown in Table 3) because they stated that sharing or using linked data was unacceptable under any conditions.

**Table 2:** Characteristics of the study sample

Characteristic	Scotland ***N*** = 1004 (%)	Sweden ***N*** = 974 (%)
*Gender*		
Male	421 (41.9%)	499 (51.2%)
Female	583 (58.1%)	475 (48.8%)
*Age*		
18–34 years	275 (27.4%)	354 (36.3%)
35–54 years	358 (35.7%)	411 (42.2%)
55+ years	371 (37.0%)	209 (21.5%)
*Employment*^a^		
Working part or full time	557 (55.5%)	627 (64.3%)
Not working	444 (44.2%)	341 (35.0%)

^a^ missing data: Scotland (*n* = 3) and Sweden (*n* = 6)

**Table 3:** Number of respondents routed-out after responding “data linkage should not be permitted under any circumstances” to the initial survey questions

Initial survey questions, in order^**a**^	Scotland (n)	Sweden (n)
Purposes of research	67	51
Who are the researchers	104	186
What types of information may be linked	135	128
Management of potential profits	145	140
Arrangements for oversight/monitoring	8	5
Public involvement in data linkage research	2	3
**Total**	**461**	**513**

^**a**^Each question asked respondents which of the levels in Table 1 was closest to their view about the attribute (or that data linkage should not be permitted)

The results of the conditional logit model (Table 4) suggested that all attribute levels were statistically significant (*p* < 0.01). The positive coefficients indicated that all levels were preferred, relative to the ‘worst’ level of each attribute that were used as a ‘base level’. The absolute values of the estimated coefficients cannot be interpreted as pure ‘preference weights’ because these estimated values measure *relative* preference [27] and, therefore, only the relative sizes of changes across levels, in each country, have meaningful interpretations.

**Table 4:** Results of the conditional logit model

Attribute and level	Estimate coefficient (standard error)		
	Scotland	Sweden	Country comparison^b^
Researchers:			
University researchers	0.214*** (0.05)	0.168** (0.05)	0.047 (0.07)
University /health service staff	0.500*** (0.05)	0.312*** (0.05)	0.188** (0.07)
University/health service staff/government	0.445*** (0.05)	0.337*** (0.05)	0.108 (0.07)
University/health service staff/government/commercial2	Base level^a^		
Data to be linked:			
Primary care linked to other health records	0.918*** (0.05)	0.706*** (0.05)	0.212** (0.07)
Health records linked to social care/education records	0.664*** (0.05)	0.403*** (0.05)	0.261*** (0.07)
Health records linked to social care/education/employment/benefits records	0.407*** (0.05)	0.171*** (0.05)	0.236** (0.07)
Health records linked to social care/education/ employment/benefits records/private sector	Base level^a^		
Purpose:			
Direct benefits for the people whose information is used	0.322*** (0.04)	0.254*** (0.04)	0.068 (0.06)
Research conducted if it will have general public benefits	0.548*** (0.04)	0.430*** (0.04)	0.118* (0.06)
Research for any reason	Base level^a^		
Profit-making:			
Nobody profits	0.326*** (0.05)	0.171*** (0.05)	0.156* (0.07)
Profit shared with the public	0.579*** (0.05)	0.397*** (0.05)	0.182* (0.07)
Profit invested into public services	0.739*** (0.05)	0.506*** (0.05)	0.233** (0.07)
Profit goes to those doing the research	Base level^a^		
Oversight:			
Overseen by independent body	0.346*** (0.05)	0.420*** (0.05)	−0.074 (0.07)
Overseen by relevant public service	0.265*** (0.05)	0.457*** (0.05)	−0.192** (0.07)
Overseen by Government	0.066 (0.05)	0.289*** (0.05)	−0.223** (0.07)
Overseen by the organisation undertaking the research	Base level^a^		
Constant	0.886*** (0.08)	0.620*** (0.08)	0.266* (0.12)
Number of observations	**18,072**	**17,532**	**35,604**

*p < 0.05; **p < 0.01; ***p < 0.001

^a^ Each attribute used categorical levels, which were dummy coded relative to a base level (Table 1) that was deemed to be the ‘worst’

^b^ The country comparison model, estimated using pooled data using a condition logit model, included interaction terms between dummy variables that identified the respondent’s nationality (1 = Scottish) and each attribute level

The positive interaction terms between levels and nationality could suggest that Scottish respondents have stronger preferences over using linked data than those living in Sweden. However, the inflated coefficients for Scotland may be driven by differences in scale (choice consistency). Table 5 shows the results of the heteroskedastic conditional logit model with interaction and scale terms. These results show that the scale term was statistically significant at the 10% level (*p* = 0.052) which can be interpreted to indicate that the Scottish sample were, on average, more ‘consistent’ in their decision making when making choices. The estimated error term (the variance of the unobservable element of utility) was smaller in the Scottish sample relative to the Swedish sample. The statistically significant and positive constant term also suggests that, all else being equal, respondents preferred their data not to be used or linked.

**Table 5:** Results of the heteroskedastic conditional logit model

Attribute and level	Estimate coefficient (standard error)		
	Pooled data (Scotland and Sweden)	Interaction terms^b^	
**Researchers:**			
University researchers	0.168** (0.05)	0.001 (0.07)	
University /health service staff	0.312*** (0.05)	0.080 (0.08)	
University/health service staff/government	0.337*** (0.05)	0.012 (0.08)	
University/health service staff/government/commercial	Base level^a^		
**Data to be linked:**			
Primary care linked to other health records	0.706*** (0.05)	0.014 (0.11)	
Health records linked to social care/education records	0.403*** (0.05)	0.118 (0.09)	
Health records linked to social care/education/employment/benefits records	0.171*** (0.05)	0.148 (0.08)	
Health records linked to social care/education/ employment/benefits records/private sector	Base level^a^		
**Purpose:**			
Direct benefits for the people whose information is used	0.253*** (0.03)	0^c^	
Research conducted if it will have general public benefits	0.430*** (0.04)	0^c^	
Research for any reason	Base level^a^		
**Profit-making:**			
Nobody profits	0.171*** (0.05)	0.086 (0.07)	
Profit shared with the public	0.397*** (0.05)	0.057 (0.08)	
Profit invested into public services	0.506*** (0.05)	0.074 (0.09)	
Profit goes to those doing the research	Base level^a^		
**Oversight:**			
Overseen by independent body	0.420*** (0.05)	−0.148* (0.07)	
Overseen by relevant public service	0.457*** (0.05)	−0.249*** (0.07)	
Overseen by Government	0.289*** (0.05)	−0.238*** (0.07)	
Overseen by the organisation undertaking the research	Base level^a^		
Constant	0.620*** (0.08)	0.076 (0.11)	
Scale term (if the respondent is Scottish)		0.242	(0.12)
**Number of observations**	**35,604**		

*p < 0.05; **p < 0.01; ***p < 0.001

^a^ Each attribute used categorical levels, which were dummy coded relative to a base level (Table 1) that was deemed to be the ‘worst’

^b^ Interaction terms indicate the effect of being Scottish on the estimated coefficients

^c^ The estimated model included an interaction term in which the attribute ‘purpose’ was restricted to zero

The attribute for the source of data being linked aligned with a priori expectations as respondents preferred fewer sources of data being linked in both countries. On average, respondents generally preferred different types of health records being linked together rather than health records linked with public social, education or employment records, although all of these scenarios were preferable to linkage with private sector records (the base case). Respondents in both countries preferred that nobody profits rather than the profits go to those carrying out the work. However, people in both countries, on average, preferred profit to be invested into public services or shared with the public than nobody profiting at all.

For the attributes ‘who does the research’, ‘type of data being linked’ and ‘profit-making’, average preferences between the two countries were relatively homogenous. Similarly, the constant term did not significantly differ between the groups indicating respondents in one country were no more or less likely to state that neither scenario was acceptable. The most prominent difference in preferences between Scotland and Sweden were for the attribute ‘oversight’ (see Table 5). Although, on average, respondents in both countries preferred external oversight of the research, the respondents living in Sweden seemed to value this as being more important, as it had a larger impact on their choice-making.

**Fig. 2:**
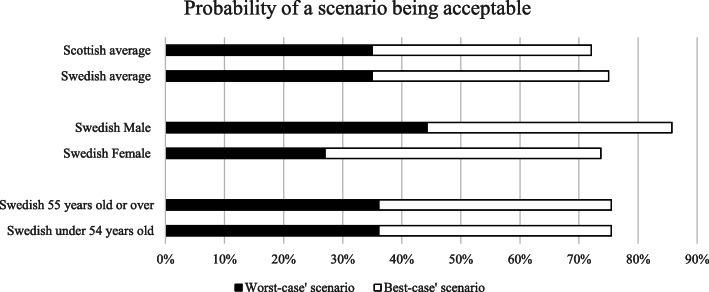
Probability of a scenario describing linked data being acceptable

For the typical data linkage scenario, the probability of acceptance, on average, was 85.7% in Sweden and 82.4% in Scotland. In the ‘best-case’ scenario of data linkage, the probability, on average, of a Swedish person accepting the alternative was estimated to be 75.0% compared with 72.1% in Scotland. In the ‘worst-case’, the probability of the scenario being acceptable on average, was 35.0% in both countries. Figure 2 shows the probability of these different scenarios being acceptable in different subgroups of the sample. Differences in acceptability of the ‘worst-case’ scenario were most prominent when considering gender, with men 50% more likely to find this case of data linkage acceptable compared to women (44 and 27% retrospectively).

An interactive model showing average probability of acceptability in different scenarios is available in the online supplementary materials (see Additional file). This allows the reader to see the impact of changing attribute levels on the average probability of acceptability of, for example, the typical scenario. Changing the research attribute to the base level, ‘research for any reason’ (and keeping all others the same) decreased the probability of acceptance of the typical scenario by 6.1 and 7.1% in Sweden and Scotland respectively. In comparison, changing only the type of data attribute to the base level of ‘health data linked to social care, education, employment and private-sector data’ decreased the probability of the scenario being accepted by 11.0 and 12.6% in the two jurisdictions.

## Discussion

This study quantified the aspects of sharing and using different types of linked data that drove the preferences of members of the public and estimated the potential impact on acceptability of using anonymised linked data. It is the first study to directly compare preferences for the sharing and use of linked data between two countries, showing that there were considerable similarities in average preferences amongst members of the public in Scotland and Sweden. The exception to these common preferences was that people living in Sweden were more influenced by who should have control of ‘external oversight’ when sharing or using linked data.

The considerable similarities between preferences in the two jurisdictions are curious, given the perceived differences in the use of linked data between the two countries. In Sweden, the use of a unique personal identifier means that the creation and use of registries of data are common [7, 8] but this is less so in Scotland. However, Scotland is distinct from the rest of the UK in that, since the 1970s, all NHS patients have been assigned a Community Health Index number which registers data on address, postcode, GP, date of birth, region of registration and date of death. This is used in all primary health care activities and hospital-based clinical information systems, throughout NHS Scotland, including the Emergency Care Summary. Nevertheless, the lack of a universal personal identifier reduces the ease with which data from different sectors can be linked. Throughout the UK, health data science research is increasing [40] and there is an increasing move towards data use and linkage across sectors where “Data will drive Scotland’s next economic revolution” [41]; knowledge of public preferences and acceptability will be vital in ensuring that there is a social license for such work [9]. Therefore, it is reassuring that the preferences in Scotland are so similar to those in a jurisdiction where data use and linkage are perceived as both commonplace and acceptable.

The main differences between the two jurisdictions related to external oversight of data use and linkage. Swedish respondents were more likely to prefer oversight by either the government or the relevant public service, or, to a lesser extent, an independent body (Table 5) than were the Scottish respondents. Swedish national data registries are tightly controlled and their use requires review both by ethics committees and the government organisation Statistics Sweden [8]. In addition, the delivery of healthcare is devolved locally and one study found that Swedes were more likely to want to be involved in local healthcare organisations then were people in England [42]. However, such oversight is not always valued in Sweden. In one study on public perceptions of biobank research, respondents did not trust either the government or county councils to evaluate the risks and benefits of genetic research being proposed [43].

The “best case” scenario chosen in this study was the most risk averse scenario. However, the average probability of this being accepted (over 70% in both jurisdictions) was less than that of the typical scenario, where the average probability of it being accepted was over 80%. This typical scenario was chosen to be similar to much of the health research conducted with linked data, such as that cited earlier [2, 3]. Previous studies have suggested that public benefit is potentially a key condition for acceptability of research using linked data [12, 44, 45]. However, changing this single attribute to the base case (‘research for any reason’) decreased the probability of acceptance of the scenario by only 6–7%. In comparison, changing only the type of data that were linked to the base level (linkage of multiple types of public and private sector data) decreased the probability of the scenario being accepted by almost twice as much. When considered individually in surveys, the purpose of the research, particularly for research involving commercial companies, has been shown to be important for public acceptability of the research [12, 44, 45]. Those studies were limited in their abilities to compare factors that influence acceptability. This study has been able to quantify the differences in acceptability between such factors and this suggests that public benefit may be slightly less important to respondents than the types of data that were being linked.

Multiple types of data linked together, as with the base case, may be of concern to respondents because this is currently an unfamiliar consideration within data linkage. Some members of the public (33%) in the UK are very aware that the NHS uses health data in research, but much fewer (16%) are aware that commercial companies also do so [44]. By extrapolation, it would be expected that even fewer would be aware of the potential for doing research using both types of data linked together. The involvement of the private sector in the use of health data alone is already known to be a controversial topic. Other research has found that 17% of the general public would not accept commercial use of data at all [44] and qualitative studies found that there is a belief in a hidden agenda with commercial companies [31]. Linkage of supermarket loyalty cards with other sources of lifestyle data has been proposed, for example, as a resource for research into obesity [46]. Therefore, there is a need to understand further these preferences of the public with regard to data linkage at such scale, before this takes place.

For both countries, the least preferred option for the profit attribute, compared to the base case, was that nobody would profit. Profit by commercial companies, particularly large amounts of profit, is known to be of particular concern to the public [13]. Many people are also concerned about the British government profiting from selling health data to private healthcare companies [47]. Nonetheless, in this study, the preferred option was that profits be reinvested into the public services, suggesting a very complex and nuanced set of opinions and preferences around the creation and use of profit from the use of health data, particularly when commercial companies are involved. This has been explored in detail by the Wellcome Trust [44] and this study adds to that evidence base by quantifying the preferences. However, it is acknowledged that this study could not tease out the differences in respondents’ preferences about how companies made the profit described in the scenarios. For example, the research mentioned in the scenario could have created profit by sales of a newly developed healthcare product or increased sales of an existing product, either of which could deliver the levels used for the attribute “the purpose of the research”.

The large numbers of respondents in both countries, and their representativeness in observable demographics to the overall national population, were key strengths of the study. These substantial numbers allowed comparisons across subgroups, showing differences in acceptability of the worst-case scenario between men and women and young and old. All respondents were recruited using an internet panel provider to facilitate collecting a large study sample relatively inexpensively. Although DCEs in healthcare are increasingly administered online [48], the limitations of using online surveys and/or internet panels for stated preference studies has not been thoroughly investigated. For instance, respondents to online DCEs are more likely to be computer-literate and, particularly in older age groups, may not be representative of the general population. Their views could possibly be different to those who would have preferred a paper-based method. However, there is some evidence suggesting online health surveys provide good quality data compared with other methods such as postal surveys or telephone interviews [49]. In addition, previous DCEs using more population-representative sampling frames (e.g. from the electoral roll) have resulted in very low response rates, and hence had limited representativeness and generalisability for different reasons [50].

The DCE was restricted to those respondents who did not believe the sharing and use of linked data should be allowed under any circumstances. The choice to limit the population in this way was made to ensure that we could investigate the nuanced opinions of those who agreed, in principle, to sharing and using linked data [30]. Had those people been included, there was a risk that those respondents would have selected the ‘opt-out’ option in every scenario. When a respondent chooses to opt-out, nothing about their preferences are revealed as there are no trade-offs with the attributes or levels presented in the hypothetical alternatives. For respondents who did not believe the sharing and use of linked data should be allowed under any circumstances, the option to opt-out may have drawn disproportionately from the other alternatives affecting the average and relative choice shares presented in Fig. 2 [51]. Table 3 shows, however, that there was not complete opposition to sharing and using linked data. Respondents were not aware that they would be routed-out of the DCE by answering in a particular way, so there is no reason to doubt their answers. In both jurisdictions, only small numbers of respondents did not accept that research should be conducted for one of the purposes presented in the scenarios (the first question). Who the researchers were and who was allowed to profit were much more controversial topics, particularly in Sweden, with most respondents being routed-out at these questions (Table 3). It is possible, therefore, that we were too risk averse in excluding these respondents. In addition, the removal of these individuals limits the generalisability of the study findings. Future research may wish to extend the study sample to include respondents who disagree with the sharing and use of linked data in principle and investigate two-way or higher-order interactions between the attribute levels by incorporating these into the experimental design [28] to understand if certain combinations result in more/less acceptable scenarios. There is also the need for future qualitative investigations in Sweden to understand why so many respondents had concerns regarding sharing and using data, given how commonplace are the use of national registries.

## Conclusion

This study suggests that the public living in Scotland and Sweden are open to using anonymised linked data in certain scenarios for research purposes but some caution is advisable if health data are linked to non-health data. Despite the use of linked data for national registries being more common in Sweden, replicating the Scottish DCE there has revealed substantial similarities in the preferences of the public in the two jurisdictions. Given the considerable investment in data intensive health research and the use of linked data in Scotland, this suggests there may be considerable value in further comparative work between the two countries. In particular, further research to understand the reasons underpinning public concerns around data linkage and sharing in relation to experiences in Sweden might be valuable to inform the development of future practices in Scotland.

### Supplementary information

**Additional file 1.** An interactive model showing average probability of acceptability in different scenarios.

## Data Availability

An interactive model has been made available as supplementary material in Appendix B.

## Appendix

Random utility theory acknowledges that there is a ‘random’ component of utility that is unobserved by the analyst, because it is due to unobservable factors or just psychological impulse [52]. Therefore an individual’s, *n*, utility, *U*, for alternative, *j*, can be defined as:

1 $$ {U}_{n,j}={V}_{n,j}+{\varepsilon}_{n,j} $$

2 $$ {V}_{n,j}={f} ({\beta}_{k}, {X}_{n,j}) $$

Where *V*_*n*, *j*_ is the observed component of utility which is a function of *β*_*k*_ (the utility associated with the *K* attributes and the level of that attribute (*X*)).

In this case, the utility function to be estimated is:

3 $$ {\displaystyle \begin{array}{c}{U}_{n\;j}={\alpha None}_{n\;j}+{\beta}_1{RUNI}_{n\;j}+{\beta}_2{RUNHS}_{n\;j}+{\beta}_3{ RUNHS G}_{n\;j}+{\beta}_4{INHS}_{n\;j}+{\beta}_5{ INHS E}_{n\;j}+\\ {}{\beta}_6{ INHS E B}_{n\;j}+{\beta}_7{BENEFITD}_{n\;j}+{\beta}_8{BENEFITG}_{n\;j}+{\beta}_9{PROFITN}_{n\;j}+{\beta}_{10}{PROFITPUB}_{n\;j}+\\ {}{\beta}_{11}{ PROFITN V}_{n\;j}+{\beta}_{12}{NONGOV}_{n\;j}+{\beta}_{13}{PUB}_{n\;j}+{\beta}_{14}{GOV}_{n\;j}+{\varepsilon}_{n\;j}\;\end{array}} $$

In Eq 3, *β*_1 − 14_ are preference weights associated with each attribute level (as defined in Table 6), relative to the base case (dropped). Note, *β*_*k*_ assumes homogenous preferences as it does not vary by *n*. The constant, *α*, reflects the utility associated with ‘opting-out’ with no data linkage. The random component, *ε*_*n*, *j*_, means *U* cannot be perfectly observed.

**Table 6:** Attribute level coding

Attributes and levels		Labels
The researchers are:	Only university researchers	RUNI
	Only university researchers or NHS staff (researchers employed by a county council)	RUNHS
	Only university researchers, NHS staff or government researchers (researchers employed by a county council or researchers employed by one of the authorities)	RUNHSG
	University researchers, NHS staff, government researchers (researchers employed by a county council or researchers employed by one of the authorities) and commercial researchers such as market research organisations or pharmaceutical companies^a^	RUNHSGP
The type of data being linked:	Information from your GP (primary care) records being linked with information from your other NHS (county council) health records e.g. hospital records	INHS
	Information from your NHS (county council) health records being linked with information from your social care or education records.	INHSE
	Information from your NHS (county council) health records being linked with information from your social care or education records, or from your employment and benefits (national health insurance) records.	INHSEB
	Information from your NHS (county council) health records being linked with information from your social care, education, employment, and benefits (national health insurance) records, as well as information collected about you in the private sector e.g. through online shopping accounts^a^	INHSEBX
The purpose of the research:	Research using linked information should only be conducted if it will have direct benefits for the people whose information is being used.	BENEFITD
	Research using linked information should only be conducted if it will have general public benefits.	BENEFITG
	Research using linked information should be allowed for any reason. ^a^	BENEFITX
Profit-Making:	Nobody should be allowed to profit from research carried out using linked information.	PROFITN
	Any profit made from research carried out using linked information should be shared with the public.	PROFITPUB
	Any profit made from research carried out using linked information should be invested into public services.	PROFITINV
	Any profit made from research carried out using linked information should be kept by those carrying out the research. ^a^	PROFITR
Oversight:	The process should be overseen by the Scottish (Swedish) Government.	NONGOV
	The process should be overseen by a non-governmental independent body (an independent body that is not part of the Swedish Government).	PUB
	The process should be overseen by the relevant public service(s); for example, research that uses information from people’s health records should be overseen by the NHS (county council).	GOV
	The process should be overseen by the organisations undertaking the research^a^.	ORG

^a^Defined as the base level for dummy coding of the categorical levels

In the heteroskedastic conditional logit model the utility function is estimated as

4 $$ {\displaystyle \begin{array}{c}{U}_{n,j}={\lambda}_n{\alpha}_1{None}_{n\;j}+{\lambda}_n{\beta}_1{RUNI}_{n\;j}+{\lambda}_n{\beta}_2{RUNHS}_{n\;j}+{\lambda}_n{\beta}_3{ RUNHS G}_{n\;j}+{\lambda}_n{\beta}_4{INHS}_{n\;j}+\\ {}{\lambda}_n{\beta}_5{ INHS E}_{n\;j}+{\lambda}_n{\beta}_6{ INHS E B}_{n\kern0em j}+{\lambda}_n{\beta}_7{BENEFITD}_{n\kern0em j}+{\lambda}_n{\beta}_8{BENEFITG}_{n\kern0em j}+\\ {}{\lambda}_n{\beta}_9{PROFITN}_{n\;j}+{\lambda}_n{\beta}_{10}{PROFITPUB}_{n\;j}+{\lambda}_n{\beta}_{11}{PROFITN V}_{n\;j}+{\lambda}_n{\beta}_{12}{NONGOV}_{n\;j}+\\ {}{\lambda}_n{\beta}_{13}{PUB}_{n\;j}{\mathrm{Soct}}_n+{\lambda}_n{\beta}_{14}{GOV}_{n\;j}+{\lambda}_n{\alpha}_2{None}_{n\;j}{\mathrm{Soct}}_n+{\lambda}_n{\beta}_{15}{RUNI}_{n\;j}{\mathrm{Soct}}_n+\\ {}{\lambda}_n{\beta}_{16}{RUNHS}_{n\;j}{\mathrm{Soct}}_n+{\lambda}_n{\beta}_{17}{RUNHS G}_{n\;j}{\mathrm{Soct}}_n+{\lambda}_n{\beta}_{18}{INHS}_{n\;j}{\mathrm{Soct}}_n+{\lambda}_n{\beta}_{19}{INHS E}_{n\;j}{\mathrm{Soct}}_n+\\ {}{\lambda}_n{\beta}_{20}{INHS E B}_{n\;j}{\mathrm{Soct}}_n+{\lambda}_n{\beta}_{21}{BENEFITD}_{n\;j}{\mathrm{Soct}}_n+{\lambda}_n{\beta}_{22}{BENEFITG}_{n\;j}{\mathrm{Soct}}_n+\\ {}{\lambda}_n{\beta}_{23}{PROFITN}_{n\;j}{\mathrm{Soct}}_n+{\lambda}_n{\beta}_{24}{PROFITPUB}_{n\;j}{\mathrm{Soct}}_n+{\lambda}_n{\beta}_{25}{PROFITN V}_{n\;j}{\mathrm{Soct}}_n+\kern0.36em \\ {}\;{\lambda}_n{\beta}_{26}{NONGOV}_{n\;j}{\mathrm{Soct}}_n+{\lambda}_n{\beta}_{27}{PUB}_{n\;j}{\mathrm{Soct}}_n+{\lambda}_n{\beta}_{28}{GOV}_{n\;j}{\mathrm{Soct}}_n+{\varepsilon}_{n\;j}\;\end{array}} $$

In Eq 4, Soct_*n*_ is a dummy variable equal to 1 when the respondent was Scottish and 0 when they were Swedish. The dummy was interacted with each of the attribute levels. Therefore *β*_15 − 28_ are the difference in preference weights associated with each attribute level due to being Scottish. Where *λ*_*n*_ is the relative scale parameter for the Scottish sample relative to the Swedish sample. HCL model parameterises *λ*_*n*_ as exp(*Scot γ*), and therefore testing the significance of *γ* is testing if individuals’ nationality had a statistically significant effect on the scale parameter.

## Abbreviations

GP: General Practitioner
DCE: Discrete choice experiment
NHS: National Health Service
UK: United Kingdom
